# Growing Old Happily

**Published:** 1860-05

**Authors:** 


### GROWING OLD HAPPILY.

THERE is naturally but one disease, that of old age. To leave the world as gently as go out the embers on the hearth, or as the candle in its socket, without pain, or shock, or spasm, this is worth taking pains for! Literally, the lot is terrible, of a man with tottering limbs, and gray hairs, dying by piece-meal from racking rheumatism, from torturing gout, or the slow-eating cancer! the mind all the while, by reason of incessant pain growing morose, querulous, bitter, and atheistic! On the other hand, how ineffably beautiful is it to arrive at a hearty, buoyant old age, without ache or pain or sadness; sunshine always in the face, gladness in the eye, the heart meanwhile welling up and running over with human sympathies and love divine, of whom "my mother sang" so often in the clear, sweet, and cheery tones of youth and health.

"The day glides swiftly o'er their head,
Made up of innocence and love,
And soft and silent as the shade,
Their nightly minutes gently move.

"Quick as their thought their joys come on,
But fly not half so swift away;
Their souls are ever bright as noon,
And calm as summer evenings be."

And when their work is done, their journey ended, the life of time melts into an immortal existence:

"As fades a summer cloud away,
As sinks a gale when storms are o'er,
As gently shuts the eye of day,
As dies a wave along the shore."

To have the lamp of life thus go out, physically, we must live regularly, temperately, actively; for by these means only can the great human clock work well until all the wheels wear out together, and all cease their running at the same instant: then there is no shock, no pain, no torture, and scarce a perceptible struggle, so that the moment of departure can be noted only by the most scrutinizing eye. Reader! may such be your exit and mine.

